# A Text-Book of Medicine

**Published:** 1887-06

**Authors:** 


*A Text-book of Medicine.* By Dr. ADOLF STRÜMPELL. Translated from the 2nd and 3rd German Editions by H. F. VICKERY, M.D., and P. C. KNAPP, M.D. Pp. 981. London: H. K. LEWIS. 1887.

English readers are indebted to American enterprise for the translation of Dr. Strümpell's admirable work. Under the editorial supervision of Dr. Frederick C. Shattuck, Instructor in the Theory and Practice of Physic at Harvard Medical School, Drs. Vickery and Knapp have done their work exceedingly well. The graceful easy-flowing English sentences give no evidence of transliteration from the cumbersome German. Dr. Shattuck's interpolated notes and remarks are valuable, and distinctly add to the usefulness of the work.

The popularity of the work in Germany is very strong evidence in its favour. So far as we have been able to judge it is admirably suited for students, being concise, clear, well arranged, and fully up to date. Special commendation must be given to the chapters on nerve-diseases. Probably nowhere else in the English language will the student find this important and ever-changing subject so well and so succinctly handled. We heartily commend the work to our readers.

